# CARTO UNIVU-Guided Micra Implantation in Ebstein Anomaly

**DOI:** 10.1016/j.jaccas.2025.103784

**Published:** 2025-06-18

**Authors:** Yuya Nakamura, Yosuke Kai, Rimpei Ueno, Yoshikazu Suzuki, Masaya Ochiai, Takamasa Ishikawa, Yuki Takai, Shuhei Arai, Taku Asano, Toshiro Shinke

**Affiliations:** Division of Cardiology, Department of Medicine, School of Medicine, Showa University, Tokyo, Japan

**Keywords:** bradycardia, congenital heart defect, Ebstein’s anomaly, electroanatomical mapping

## Abstract

**Background:**

Ebstein anomaly (EA) is a rare congenital heart defect characterized by tricuspid valve malformation and severe tricuspid regurgitation. This poses challenges for conventional pacemaker implantation.

**Case Summary:**

An 86-year-old man with EA, atrial fibrillation, complete atrioventricular block, and heart failure underwent Micra VR leadless pacemaker implantation. The right ventricular angiography was inadequate due to torrential tricuspid regurgitation, necessitating the use of advanced mapping technologies. CARTO electroanatomical mapping and CARTO UNIVU navigation systems facilitated precise placement in the midseptal apex. At 6 months, the pacing parameters remained stable, and the patient was symptom free.

**Discussion:**

This case illustrates how advanced mapping technologies combined with leadless pacemakers can address anatomical challenges in EA. It underscores the importance of integrating modern technologies for precise device placement and improved outcomes in complex congenital conditions.

## Graphical Abstract

**Figure:**
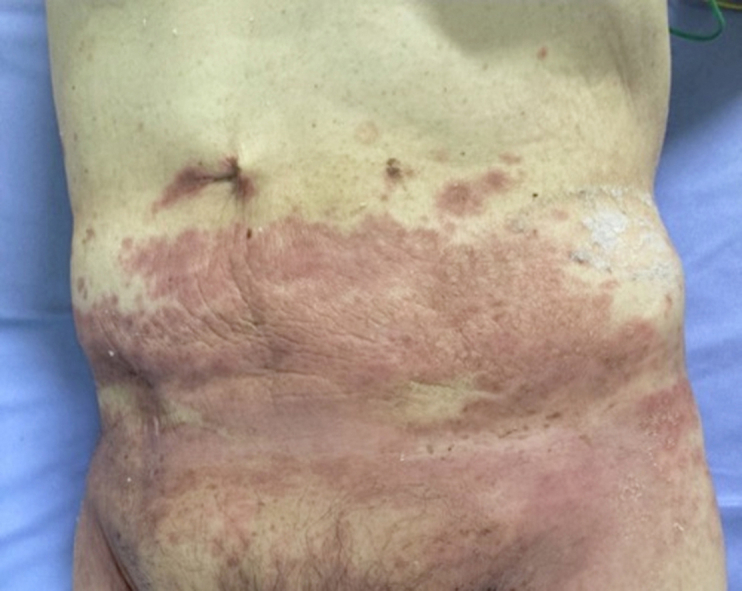

Ebstein anomaly (EA) is a rare congenital heart defect that accounts for fewer than 1% of all congenital heart diseases. It is characterized by apical displacement of the tricuspid valve leaflets, atrialization of the right ventricle (RV), and severe tricuspid regurgitation (TR).^1^ These structural anomalies result in the formation of an atrialized right ventricle (ARV) and a significantly reduced functional right ventricle (FRV), posing challenges for the implantation of traditional pacemakers.

**Take-Home Messages**

• Leadless pacemakers provide a viable alternative for managing bradycardia in patients with Ebstein anomaly.
• Advanced mapping technologies can optimize device placement in challenging anatomies like Ebstein anomaly.

Transvenous pacemaker systems pose significant risks in this population, including the potential for worsening TR and other lead-related complications due to anatomical distortion. The Micra VR leadless pacemaker (Medtronic), which eliminates the need for transvenous leads or a subcutaneous generator, offers a promising alternative for patients with complex anatomy or contraindications to conventional devices.^2^

This report describes the first case of CARTO UNIVU (Biosense Webster)–guided Micra implantation in an EA patient, highlighting the use of advanced mapping technologies to address unique anatomical challenges.

## Case Presentation

### History of Presentation

An 86-year-old man with EA and permanent atrial fibrillation presented with bradycardia, dyspnea, and lower extremity edema. These symptoms had progressively worsened over the preceding month, significantly impairing his exercise tolerance and daily activities.

### Past Medical History

**Figure 1:**
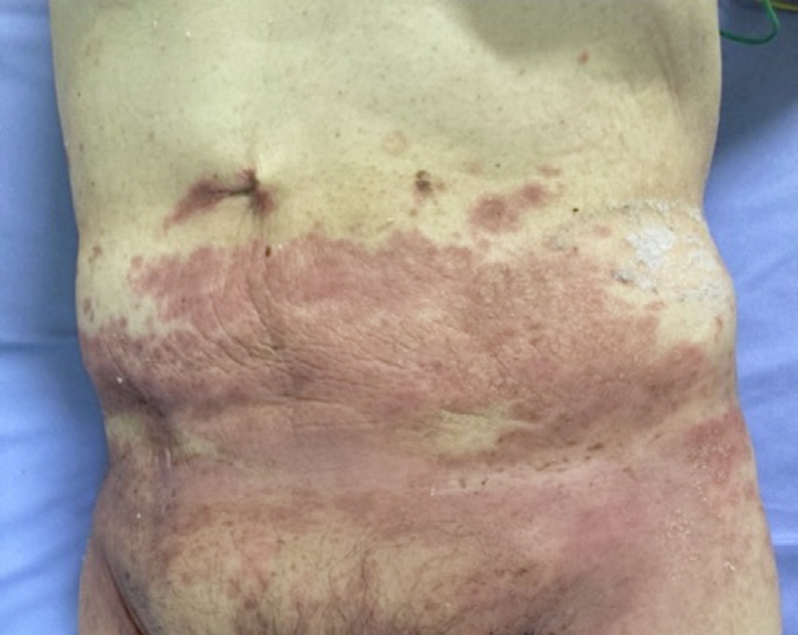
Clinical Manifestation of Metal Allergy A photograph showing erythema and localized inflammation on the patient’s lower abdomen caused by contact with a metal belt buckle. This severe allergic reaction to cobalt and chromium influenced the decision to use a leadless pacemaker, eliminating the risk of further allergic complications.

EA was diagnosed in the patient’s early adulthood, and the condition had been conservatively managed for decades. His medical history included cobalt and chromium allergies confirmed by patch testing (Figure 1). He also had comorbidities of hypertension and diabetes mellitus, both managed with oral medications. Additionally, he was on anticoagulation therapy with warfarin for atrial fibrillation.

### Diagnostic Assessment

**Figure 2:**
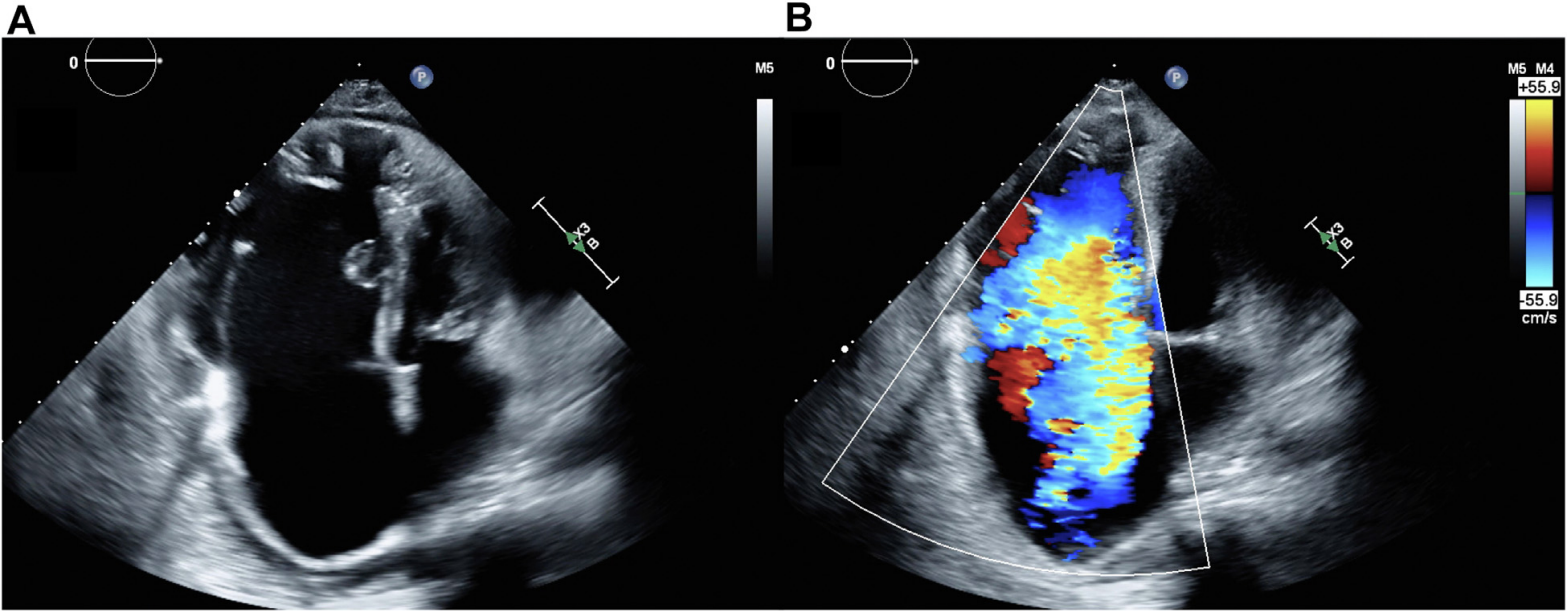
Echocardiographic Findings in Ebstein’s Anomaly Apical 4-chamber echocardiographic view illustrating apical displacement of the tricuspid valve leaflets and torrential tricuspid regurgitation, shown (A) without and (B) with color Doppler flow.

A 12-lead electrocardiogram revealed atrial fibrillation with complete atrioventricular block and a ventricular rate of 34 beats/min. Transthoracic echocardiography identified EA with apical displacement of the tricuspid valve leaflets by 33 mm/m^2^ alongside torrential TR and mild RV systolic dysfunction (Figure 2). Left ventricular function was preserved with an ejection fraction of 62%, and the left ventricular end-diastolic diameter was 48 mm. The RV function parameters included a fractional area change of 29%, tricuspid annular plane systolic excursion of 21.9 mm, and RV systolic peak velocity of 14.0 cm/s. Torrential TR resulted in an underestimated RV systolic pressure of 38 mm Hg.

## Management and Intervention

Temporary pacing was considered but deemed inappropriate due to the patient’s metal allergies. Conventional transvenous and epicardial pacemakers were also avoided because of potential allergic reactions and the risk of long-term complications such as fistula formation. The Micra VR leadless pacemaker was selected for this case due to its parylene-coated titanium construction, which minimizes direct metal exposure. On the second hospital day, the Micra VR leadless pacemaker was implanted while uninterrupted anticoagulation therapy with warfarin for atrial fibrillation was maintained.

During the procedure, RV angiography using a pigtail catheter failed to adequately delineate the chamber due to torrential TR. Given the significant structural changes in the RV associated with EA, electroanatomical mapping (EAM) was performed using the CARTO system to accurately identify specific regions within the RV. Bipolar potentials were recorded with the Octaray catheter (Biosense Webster) to construct a voltage map during atrial fibrillation with complete atrioventricular block. Only the potentials corresponding to the QRS phase were annotated for mapping to ensure accurate identification of ventricular myocardial activity. Low-voltage areas were defined as <0.1 mV, high-voltage areas as ≥0.5 mV, and areas between these thresholds were classified as the border zone.

**Figure 3:**
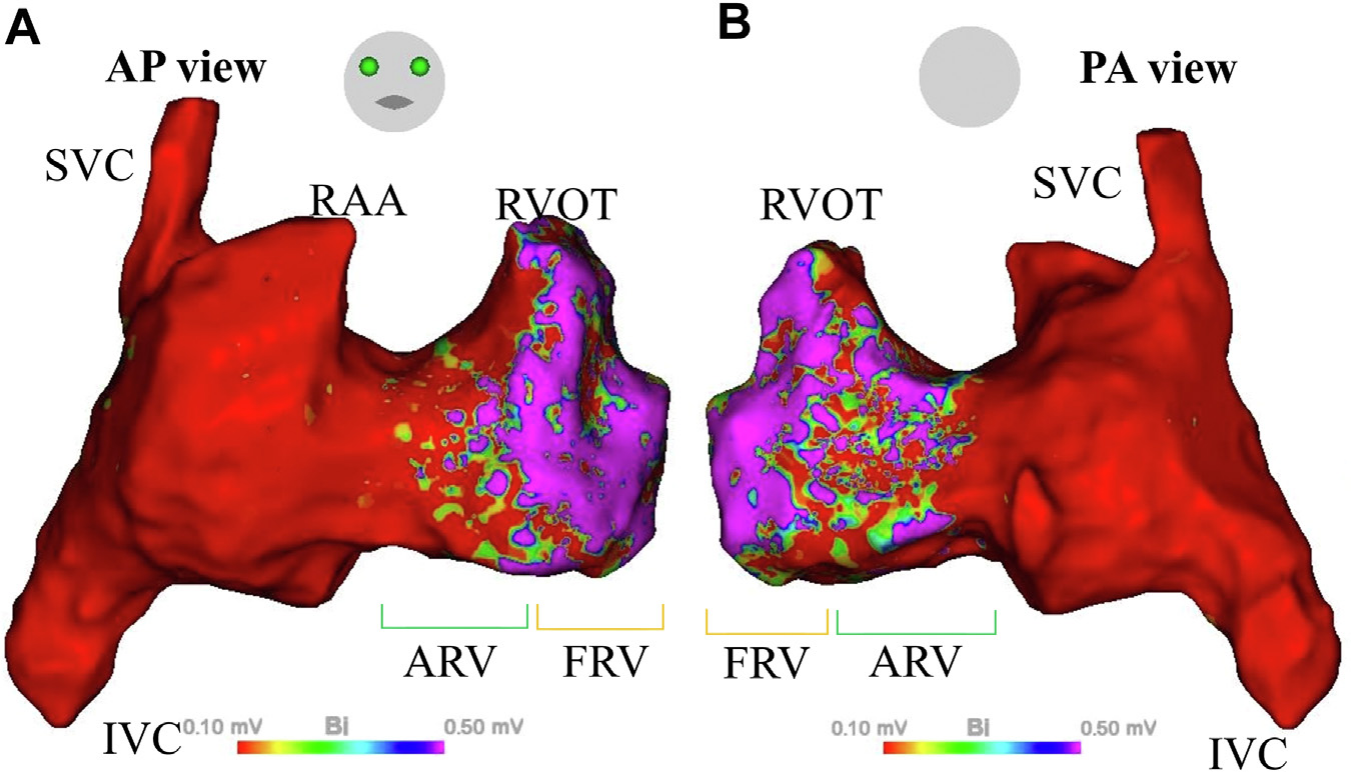
Voltage Map of the Right Heart in Ebstein Anomaly A high-density voltage map of the right heart in a patient with EA. (A) AP view and (B) PA view. Low-voltage areas (<0.1 mV), border zones (0.1-0.5 mV), and high-voltage areas (≥0.5 mV) are color coded. The voltage amplitudes may suggest a distinction between the ARV, located in the basal portion of the RV, and the FRV, situated in the apical portion. AP = anteroposterior; ARV = atrialized right ventricle; EA = Ebstein anomaly; FRV = functional right ventricle; IVC = inferior vena cava; PA = posteroanterior; RAA = right atrial appendage; RV = right ventricle; RVOT = right ventricular outflow tract; SVC = superior vena cava.

**Figure 4:**
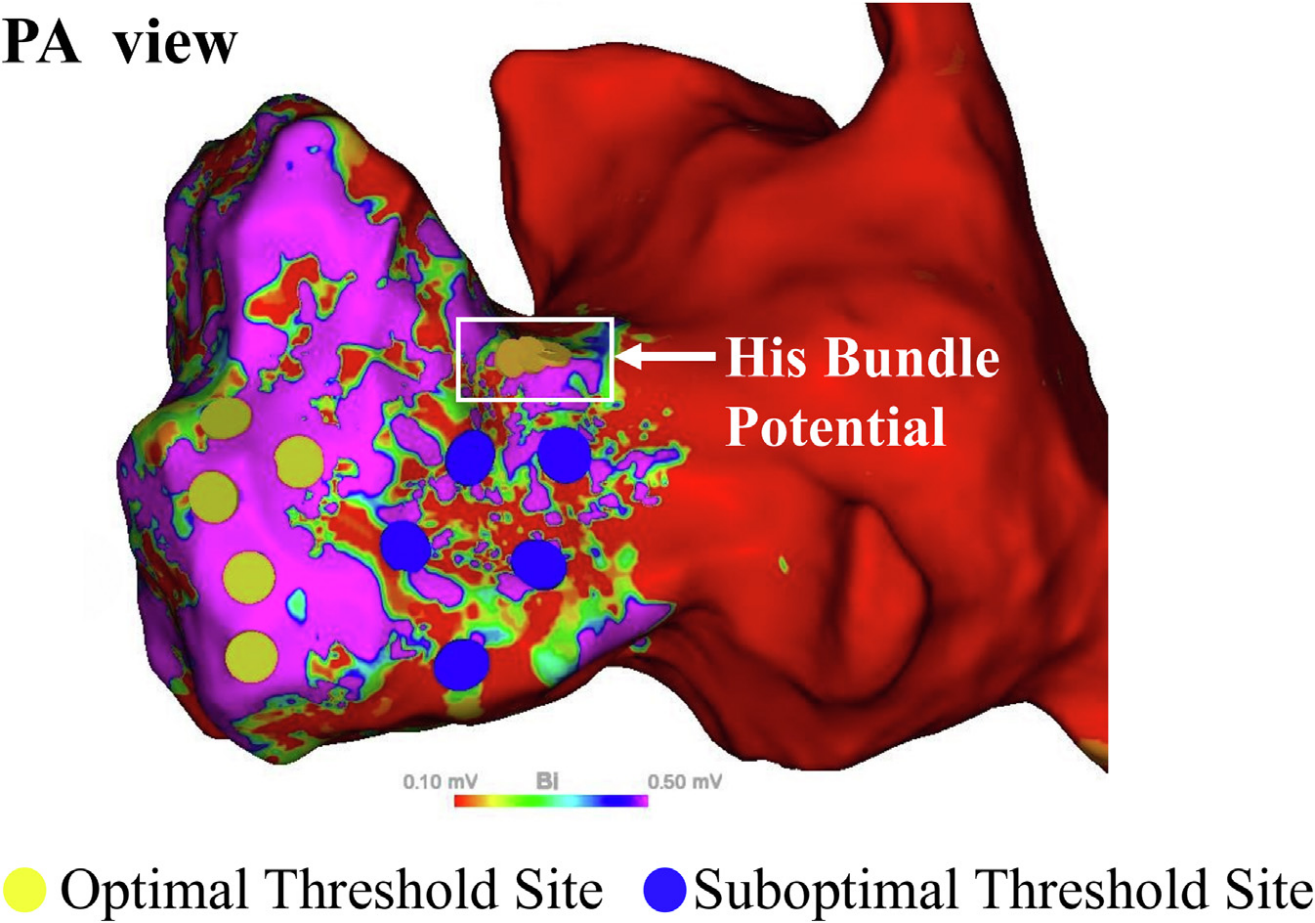
Enhanced Voltage Map With Pacing Points and His Bundle Potential in Ebstein Anomaly A voltage map of the right heart in a patient with EA, showing 5 suboptimal threshold sites (in the border zone: 0.1-0.5 mV), 5 optimal threshold sites (in the high-voltage area: ≥0.5 mV), and 1 site recording the His bundle potential. The map underscores the importance of precise mapping and site selection in achieving optimal pacing thresholds, particularly in the context of the unique anatomy and electrophysiology of EA. Abbreviations as in Figure 3.

The mapping revealed that the overall voltage in the RV was lower than that of a normal RV. The low-voltage areas and the border zone were predominantly located toward the basal side of the RV, consistent with the localization of the ARV, as commonly described in EA. Conversely, the high-voltage areas were positioned toward the apical side of the RV, corresponding to the FRV (Figure 3). Pacing evaluations further supported this hypothesis: all 5 pacing points in the border zone were identified as suboptimal threshold sites, requiring more than 2 V or failing to achieve capture. By contrast, all 5 pacing points in the high-voltage area were identified as optimal threshold sites, with thresholds of <2 V (1.2 ± 0.3 V). This detailed mapping not only facilitated the precise identification of viable myocardial regions for device implantation (Figure 4) but also validated the distinction between the ARV and FRV based on their voltage characteristics and pacing outcomes.

**Figure 5:**
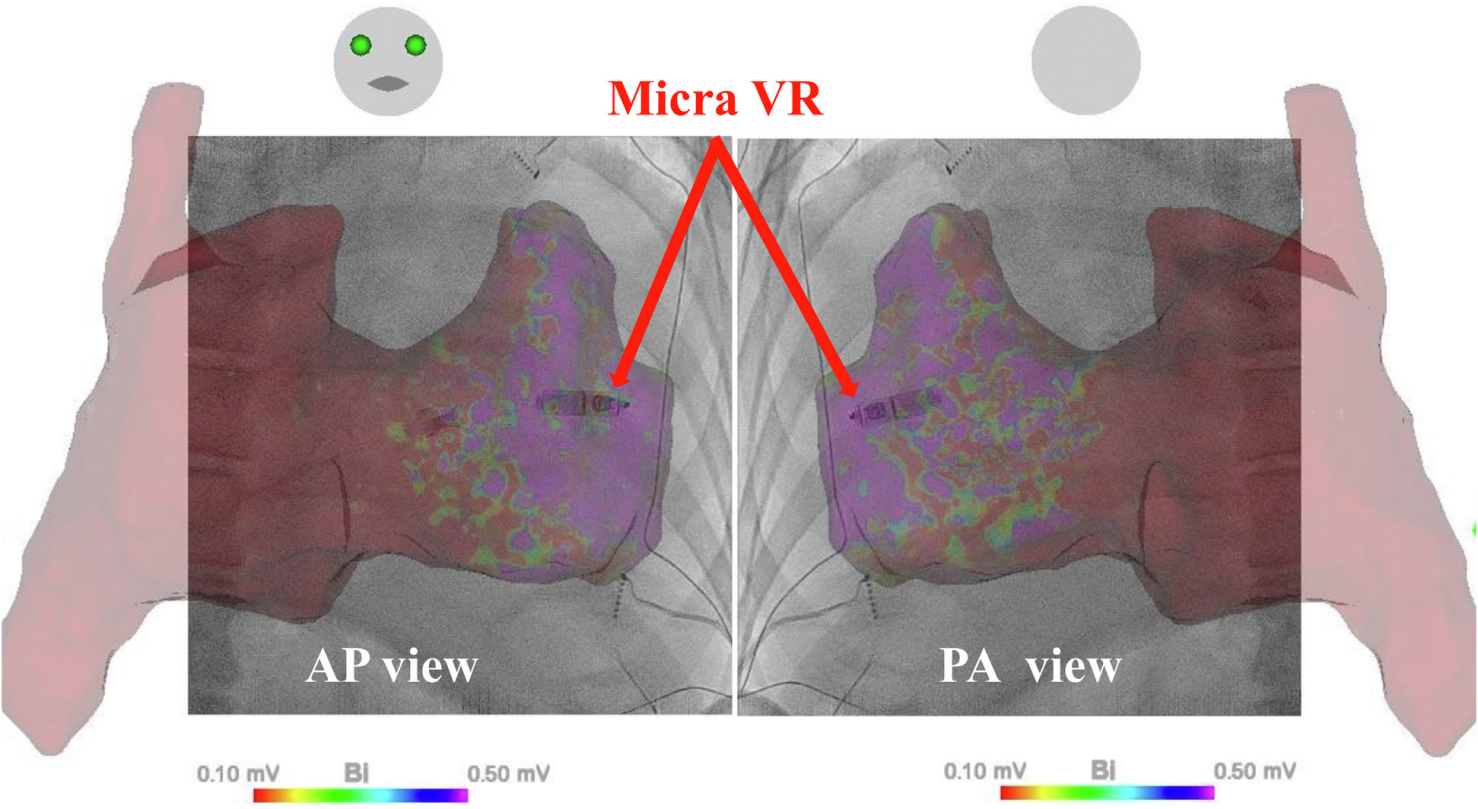
CARTO UNIVU Integration of Voltage Map and Fluoroscopic Imaging An image illustrating the integration of real-time fluoroscopic imaging with a high-density voltage map using the CARTO UNIVU system during Micra leadless pacemaker placement. This enabled precise deployment at the optimal threshold site (≥0.5 mV), minimizing risks and enhancing procedural accuracy. Abbreviations as in Figure 3.

The Micra device was deployed in the midseptal apex within the high-voltage area, corresponding to the FRV, using CARTO UNIVU navigation. The first 2 deployment attempts resulted in suboptimal fixation, with only 1 tine securing properly. To overcome this challenge, the delivery catheter was reshaped into a larger curve to better adapt to the unique atrial and ventricular morphology characteristic of EA. This adjustment allowed for precise placement at the optimal pacing site, achieving secure fixation and optimal pacing thresholds on the third attempt (Figure 5).

The pacing threshold was 0.75 V (0.24 ms), with an R-wave amplitude of 6.2 mV and an impedance of 690 Ω. The total procedure time was 115 minutes, including 19 minutes of fluoroscopy, resulting in a radiation dose of 28 Gray square centimeters (Gy·cm^2^).

## Postoperative Follow-Up

The Micra device was initially programmed to VVI mode with a set rate of 60 beats/min. After we had confirmed the absence of any issues at the catheter insertion site, the patient was mobilized on the first postoperative day. After ambulation, the rate response was activated, resulting in a significant improvement in preoperative symptoms, including bradycardia and dyspnea. The patient was carefully monitored for allergic reactions, particularly skin symptoms, with no evidence of exacerbation or other allergic complications observed during the hospital stay. As part of the postoperative management, β-blockers and sodium-glucose cotransporter 2 (SGLT2) inhibitors were initiated to address cardiovascular and metabolic conditions. The patient was discharged in stable condition on the fifth postoperative day.

At the 6-month follow-up evaluation, the patient’s pacing parameters remained stable, with a pacing threshold of 1.0 V (0.24 ms), R-wave amplitude of 6.4 mV, and impedance of 620 Ω. The ventricular pacing rate was recorded at 93%. RV function parameters—including a fractional area change of 27%, tricuspid annular plane systolic excursion of 20.5 mm, and RV systolic peak velocity of 13.5 cm/s, and left ventricular EF of 60%—showed no significant changes compared with the preimplantation measurements. Additionally, the severity of TR remained unchanged. Despite the high ventricular pacing rate, no recurrence of symptoms or significant echocardiographic change was observed.

## Discussion

This case illustrates the challenges of leadless pacemaker implantation in EA, a rare congenital heart disease with significant RV abnormalities. By integrating EAM and pacing studies, functionally viable regions of the RV were precisely identified, targeting high-voltage areas (≥0.5 mV) associated with the FRV. This approach minimized procedural risks, ensured optimal pacing thresholds, and, with CARTO UNIVU’s real-time fluoroscopic imaging, improved deployment accuracy and efficiency.

### Innovative Integration of Anatomical, Electrophysiological, and Fluoroscopic Insights for Leadless Pacemaker Implantation

The use of EAM for leadless pacemaker implantation has been documented in challenging anatomical conditions, such as sinus venosus atrial septal defects and Fontan circulation.^3^^,^^4^ These reports highlight the value of EAM in addressing structural complexities and guiding precise device placement. However, prior studies have not extensively explored the integration of electrophysiological assessments, such as pacing studies, to enhance the identification of functionally viable regions for implantation. Our case provides additional insights by integrating EAM with detailed pacing studies to assess both anatomical and electrophysiological properties of the RV. This combination allowed us to differentiate between the FRV and ARV in a patient with EA. Importantly, targeting the FRV enabled optimal pacing thresholds while avoiding complications such as myocardial injury or ineffective pacing associated with the ARV.

Moreover, the use of CARTO UNIVU to integrate fluoroscopic imaging in real-time enhanced the procedural accuracy. This approach reduced the number of deployment attempts to 3 and minimized the patient’s radiation exposure to 28 Gy·cm^2^ over 19 minutes of fluoroscopy, demonstrating procedural efficiency.

### Application of This Method to Other Devices

The integration of EAM, pacing studies, and CARTO UNIVU can be applied to various pacemaker devices,^5^ including Micra and Aveir (Abbott). In this particular case, the RV inferior wall, potentially favorable for Aveir due to its screw-in fixation mechanism, corresponded to a border zone with suboptimal pacing parameters.^6^ Conversely, the midseptal apical region, identified as the optimal pacing site, was more suitable for Micra implantation. These findings highlight the importance of preprocedural electrophysiological assessment to guide device selection, suggesting that personalized evaluation of myocardial viability may optimize clinical outcomes.

### Limitations and Future Directions

Leadless pacemakers offer significant advantages for patients with distorted anatomy or contraindications to transvenous systems, particularly in EA. By avoiding such risks as lead dislodgement, infections, and worsening TR, they address specific challenges. However, the anatomical variations in EA make standardizing voltage mapping parameters difficult. Further studies are needed to validate reproducibility and evaluate long-term outcomes.

## Conclusions

EAM combined with electrophysiological evaluation and the CARTO UNIVU navigation system represents a safe and effective strategy for leadless pacemaker implantation in patients with EA. This approach successfully addresses the unique anatomical challenges of EA, ensuring precise device placement, minimizing procedural risks, and achieving favorable outcomes.

## Funding Support and Author Disclosures

The authors have reported that they have no relationships relevant to the contents of this paper to disclose.
